# Global hotspots for coastal ecosystem-based adaptation

**DOI:** 10.1371/journal.pone.0233005

**Published:** 2020-05-29

**Authors:** Holly P. Jones, Barry Nickel, Tanja Srebotnjak, Will Turner, Mariano Gonzalez-Roglich, Erika Zavaleta, David G. Hole

**Affiliations:** 1 Department of Biological Sciences and Institute for the Study of the Environment, Sustainability, and Energy, Northern Illinois University, DeKalb, IL, United States of America; 2 Environmental Studies Department, Center for Integrated Spatial Research, University of California, Santa Cruz, CA, United States of America; 3 Hixon Center for Sustainable Environmental Design, Harvey Mudd College, Claremont, CA, United States of America; 4 Global Strategy Division, Conservation International, Arlington, Virginia, United States of America; 5 Betty and Gordon Moore Center for Science, Conservation International, Arlington, Virginia, United States of America; 6 Ecology and Evolutionary Biology, University of California, Santa Cruz, CA, United States of America; Universitat Autonoma de Barcelona, SPAIN

## Abstract

Helping the world’s coastal communities adapt to climate change impacts requires evaluating the vulnerability of coastal communities and assessing adaptation options. This includes understanding the potential for ‘natural’ infrastructure (ecosystems and the biodiversity that underpins them) to reduce communities’ vulnerability, alongside more traditional ‘hard’ infrastructure approaches. Here we present a spatially explicit global evaluation of the vulnerability of coastal-dwelling human populations to key climate change exposures and explore the potential for coastal ecosystems to help people adapt to climate change (ecosystem-based adaptation (EbA)). We find that mangroves and coral reefs are particularly well situated to help people cope with current weather extremes, a function that will only increase in importance as people adapt to climate change now and in coming decades. We find that around 30.9 million people living within 2km of the coast are highly vulnerable to tropical storms and sea-level rise (SLR). Mangroves and coral reefs overlap these threats to at least 5.3 and 3.4 million people, respectively, with substantial potential to dissipate storm surges and improve resilience against SLR effects. Significant co-benefits from mangroves also accrue, with 896 million metric tons of carbon stored in their soils and above- and below-ground biomass. Our framework offers a tool for prioritizing ‘hotspots’ of coastal EbA potential for further, national and local analyses to quantify risk reduction and, thereby, guide investment in coastal ecosystems to help people adapt to climate change. In doing so, it underscores the global role that conserving and restoring ecosystems can play in protecting human lives and livelihoods, as well as biodiversity, in the face of climate change.

## Introduction

Adapting to climate change is one of the biggest challenges facing humanity over coming decades. Its impacts are likely to be particularly acute in the coastal zone, where dense human populations, high ecological diversity, and exceptional biophysical complexity converge [1]. Many of the world’s coastal zones already bear the brunt of extreme weather, with the human toll of individual events in the past five years, such as Typhoons Bopha and Haiyan in the Philippines and Hurricanes Maria, Harvey, and Irma in the US, taking thousands of lives [2,3], and generating financial costs running into hundreds of billions of dollars in insurance, rebuilding, and repairs [4,5]. For example, Hurricane Harvey was the 2^nd^ costliest storm in the U.S. (after Hurricane Katrina), at US$100–125 billion in damages [5] and Typhoon Haiyan’s death toll was around 10,000 lives [3]. Yet around the world, population growth and economic development continue to increase the amount and value of assets in these vulnerable zones, trends that are projected to last for decades [6–8], amplifying impacts and the costs of recovery. Social or economic inequalities meanwhile often place the poorest members of society, with the least capacity to cope, in some of the most sensitive and exposed localities [7,9]. Hence, loss of lives and assets in coastal zones are likely to increase significantly in the coming decades as a result of these demographic and socio-economic trends alone, leading to a doubling or more of hurricane damages by 2100 [10,11].

Projected sea level rise (0.26–0.98m by 2100) will further exacerbate storm impacts through accelerated coastal flooding and saltwater intrusion [12,13], and threatens the very existence of low-lying island nations [1]. These trends underscore the urgent need for progress on global climate change adaptation efforts [14], with coastal protection at the fore. Traditionally, risks to coastal communities or assets have been reduced primarily through built infrastructure (e.g. sea walls, levees, beach nourishment). Ecosystem-based adaptation (EbA) approaches harness the capacity of natural ecosystems to buffer human communities against the adverse impacts of climate change through the sustainable delivery of ecosystem services that reduce risk in the face of one or more climate change threats, termed ‘adaptation services’ [15], or more generally as ‘nature-based solutions’ to climate change [16]. While EbA approaches are specifically climate change adaptation solutions, ecosystems can also be useful reducing other risks and disasters, regardless of whether they are related to climate change. Usually deployed in the form of targeted management, conservation, or restoration activities, EbA are rapidly gaining the attention of decision-makers as effective, broadly applicable and affordable options, in many adaptation contexts [17]. EbA can complement [18,19], or in some cases replace hard infrastructure, while helping to conserve natural ecosystems and the broad range of co-benefits that result (e.g., carbon storage and sequestration, habitat for commercially important fisheries, and tourism and recreation) [15,20].

Increasing evidence supports the significant biophysical role of a variety of coastal ecosystems in reducing the vulnerability of human communities to both current extreme weather events and projected future climate change driven threats [17,21–25]. For example, coastal ecosystems reduce the proportion of vulnerable people and infrastructure along exposed U.S. coastlines by around half [17]. This underscores the potential for a 100m wide coastal strip of mangroves to reduce wave heights by up to two-thirds [26], depending on the biophysical context, through their absorption of wave energy [27–29]. Coral reefs meanwhile may buffer wave energy by up to 97% in some contexts [26], significantly reducing erosion [30–32], and cutting flood damage costs in half annually [33] while wetlands, estuaries, and seagrasses trap sediments and build soils, thereby maintaining coastal elevation, preventing saltwater intrusion, and buffering water flow [23]. Indeed, wetlands in Louisiana attenuated waves from category 5 Hurricane Rita at rates as high as 25cm of wave height per kilometer [22]. A combination of these ecosystems has even greater aggregate protective potential [34]. Importantly however, the ability of coastal vegetation to buffer storm surges, wind energy, and flooding is context-specific, varying with ecosystem spatial and temporal heterogeneity, species morphology, and coastal land-use, among other factors [21,29,35]. Yet conserving or restoring coastal ecosystems is increasingly seen as an important strategy to help protect coastal communities from projected sea level rise under climate change and from more severe tropical storms [36,37].

A key advantage of EbA as an adaptation option is the retention (if an ecosystem is conserved as part of an EbA project) or production (if an ecosystem is restored) of a range of ecosystem functions and services that are provided along with the targeted adaptation service–often referred to as ‘co-benefits’ of EbA [15,38]. For example, mangrove conservation or restoration carried out to harness its coastal protection service can also maintain or provide critical nursery habitat for local fisheries, as well as timber for fuelwood and building if harvested sustainably [37]. Other co-benefits include carbon sequestration in mangroves [39], and fish production and tourism in coral reefs [38].

Despite a growing interest in EbA from the World Bank and other multi-lateral funding agencies, international conservation and development NGOs, and among the policy discussions central to decision-making within key international conventions (e.g., the Nairobi Work Program of the UNFCCC; the Sendai Framework for Disaster Risk Reduction; the Convention on Biological Diversity), the global potential for EbA options generally, and coastal EbA specifically, has not been assessed, nor integrated with information about the co-benefits of protecting those ecosystems, the relative threat of loss or degradation of those ecosystems, and to what extent they are already protected. This lack of knowledge hampers our ability to understand the global viability of coastal EbA solutions, to prioritize regions for further research or for scaling up from local pilot projects, or to distribute adaptation finance effectively. In order to explore the global potential for coastal EbA and identify likely EbA ‘hotspots’ for further research and investment, here we assess: 1) the relative vulnerability of coastal populations to climate change-driven threats from tropical storms and sea-level-rise (Vulnerability); 2) which of those vulnerable populations are likely to receive some degree of risk reduction from adjacent mangrove and coral reef ecosystems (EbA hotspots); 3) carbon storage as an example of the potential co-benefits of conserving mangroves for their adaptation role (Carbon co-benefits); and 4) the current degradation level and protection status of those critical ecosystems (Ecosystem degradation/protection).

## Methods

### Vulnerability

We used the Intergovernmental Panel on Climate Change (IPCC) Third Assessment Report’s definition of vulnerability [40], assessing coastal communities’ vulnerability to relevant climate change impacts as a function of their exposure, sensitivity, and adaptive capacity (Table 1). The most vulnerable coastal areas are those where biophysical exposure (the nature and degree to which a system experiences physical/chemical climate change induced stress) to potential climate change impacts is highest; the sensitivity of human communities (the degree to which populations are modified or affected by the effects of climate change) is greatest; and where those communities’ adaptive capacity (the ability of society to adjust or accommodate variability from climate change behaviorally and/or technologically) is lowest. To characterize vulnerability to climate change impacts in the coastal zone globally, we scaled each data layer to a 10-minute (~20 km) resolution (hereafter, ‘grid cell’), normalized exposure, sensitivity, and adaptive capacity indices to the range [0,1]. While we recognize there are a variety of formulas researchers can use to calculate vulnerability, we calculated the mean value for all relevant coastal grid cells according to the function: VU = (E+S+(1 –AC))/3, where VU is vulnerability, E is exposure, S is sensitivity, and AC is adaptive capacity.

**Table 1:** Vulnerability elements, indicators, and sources. Indicators are listed in parentheses.

Vulnerability Elements	Indicators	Source
**Exposure**	Sea-level rise	Center for Remote Sensing of Ice Sheets [41]
	Tropical storms	International Best Track Archive for Climate Stewardship [42]
**Sensitivity**	Human population density	LandScan [43]
**Adaptive Capacity**	Governance (World Governance Indicators)	Worldwide Governance Indicators [44]
	Income (Sub-national Gross Domestic Product)	Geographically-based Economic Data [45]
	Education (Net enrollment rates)	World Bank [46]
	Health (Malnourished children < 5)	Center for International Earth Science Network [47]
	Access to markets (Travel time to nearest city)	European Joint Commission Research Centre [48]
	Infrastructure (Impervious surfaces)	Rivers in Crisis [49]

The exposure sub-index was calculated as the weighted average of the standardized measures for storm frequency and SLR with storms weighted 67% and SLR 33% (see *Integrating Exposure Layers* in S1 Text). We weighted tropical storms two times higher than SLR in our assessment since tropical storms are a more immediate and acute exposure than the more protracted risks posed by sea level rise, and because there is more robust evidence for the biophysical role of mangroves and coral reefs in reducing storm- and wind-driven waves than for ameliorating SLR. We recognize that this weighting may seem arbitrary; our goal was to weight storms higher than SLR generally because they impact ecosystems in strong, short bursts repeatedly. We did a sensitivity analysis (See S1 Text) to ensure the results of this weighting captured the acute nature of tropical storms. We avoided using a more complex nonlinear function relating tropical storms to SLR in the absence of robust global data to parameterize that function. The sensitivity sub-index was determined from the standardized low-elevation coastal zone (LECZ; see S1 Text), which uses elevation, population size, and distance from coasts as indicators of sensitivity. In the adaptability sub-index, the standardized input metrics (impervious surfaces–indicators of ability to build infrastructure, travel, income, education, governance and health) were averaged with equal weight.

Finally, the full vulnerability index was calculated as the weighted average of the standardized sub-indices with adaptive capacity given double the weight of exposure and sensitivity to ensure that all components of E, S and AC contributed equally to the final VU index. If the LECZ variable in an analysis cell was missing or zero, VU was not calculated. We did not calculate VU for any cell having a LECZ variable missing or zero, and omitted any pixels with missing values. Sensitivity analyses to investigate how transformations impacted the results are discussed in S1 Text.

### EbA hotspots

To determine where EbA may represent a viable adaptation option for reducing climate change driven vulnerability of human populations in the coastal zone we used global spatially explicit datasets defining the location and spatial extent of our two key ecosystem types: mangroves from the year 2000 [50] and coral reefs for the year 2009 [51]. Although a newer mangrove dataset has become available since we began our analysis [52], we compared ours with the newer one and found <1% difference in extent, so chose to use [49]. At a global scale and at our working resolution of 10 minutes, it was not viable to compute the biophysical functions linking either ecosystem type to risk reduction. Such functions would need to be based on multiple site-specific variables ranging from bathymetry to leaf area index of the mangroves species and ecosystem width, all operating at spatial scales in the order of meters. Instead, we seek to identify ‘hotspots’ of EbA *potential* globally–i.e. regions where there is a high potential for relevant ecosystems to provide adaptation services in support of reducing human vulnerability to climate change impacts, relative to other localities. These regions then represent priorities for targeting the fine-scale, site-specific research necessary for quantifying the actual risk reduction role conferred by these ecosystems, that is critical for on-the-ground planning and management.

To determine EbA hotspots, we overlaid our spatially explicit vulnerability metric with each global ecosystem dataset. When reporting numbers of vulnerable people in the main text, we report numbers only for cells in the top 10th percentile of vulnerability within 2 km of the coast (a conservative threshold because we are counting the fewest numbers of people noted by our analysis). However, we report numbers of people in the top 20th and 50th percentiles within both 2 km and 25 km of the coast in S2 Table, since our vulnerability metric is relative (i.e. it highlights relative vulnerability comparatively to other geographies across the globe) and hence people may still be receiving a benefit without being in the highest vulnerability percentile. We consider a range from 2km to 25km because there is evidence of measurable tropical storm impacts, especially from flooding and wind damage for hundreds kilometers inland, with the most severe impacts noted closest to shore [53–55].

When we report numbers of people that intersect with ecosystems conferring potential adaptation benefits, we follow similar reasoning. We considered a range of mangrove and coral reef extent needed to confer potential adaptation benefits (5 ha– 1250 ha for mangroves; 10 ha– 2500 ha for coral reefs) because there is no consensus on how much area of ecosystem is needed to confer adaption benefits, but good information that larger areas confer greater benefits, with ecosystem topography, structure, and shape also playing important roles [32,56–59]. For mangrove ecosystems, we assumed that any of the top 10th percentile of vulnerable coastal cells within 2 km of the coast that intersect with a mangrove extent greater than 5 ha potentially receive some level of adaptation service (reported in S2 Table; a liberal threshold). But in the results, we only report numbers of people receiving an adaptation benefit in the top 10^th^ percentile of vulnerable cells within 2 km of the coast that overlap with mangrove extent of at least 1250 ha (Table 2). Similarly, for coral reef ecosystems, we assumed that any of the top 10^th^ percentile vulnerable coastal cells within 2 km of the coast that either intersect with, or are within one analysis cell of a coral reef pixel (to reflect the offshore location of coral reefs) that has at least 10 ha of coral reef is potentially receiving some level of adaptation service (reported in S2 Table, a liberal threshold). However, we only report numbers of people receiving an adaptation benefit in the top 10^th^ percentile of vulnerable cells within 2km of the coast that intersect or are within one analysis cell of a coral reef pixel that has at least 2500 ha of coral reef (Table 2). We thus define EbA hotspots as cells in the top 10^th^ percentile for vulnerability within 2km of the coast that have at least (or are adjacent to cells that have) 1250ha or 2500ha of extent of either mangroves or coral reefs, respectively.

**Table 2:** Numbers of people and extent of ecosystems in EbA hotspots.

VULNERABILITY		
**Number of people ≤2 km from coast of top 10th percentile of vulnerability for 10 min cells (highly vulnerable cells)**	**Number of People**	
Exposure + Sensitivity	109,258,529	
Vulnerability (Exposure + Sensitivity + Adaptive Capacity)	30,935,516	
**CORAL REEF—highly vulnerable cells with or adjacent to at least 2500 ha of coral reefs**		
**Highly vulnerable cells ≤ 2km from coast**	**Number of People**	**Ecosystem extent (hectares)**
Exposure + Sensitivity + coral reefs	4,963,928	846,728
Vulnerability + coral reefs	3,496,117	1,840,132
**MANGROVE—highly vulnerable cells with at least 1250 ha of mangroves**		
**Highly vulnerable cells ≤ 2km from coast**	**Number of People**	**Ecosystem extent (hectares)**
Exposure + Sensitivity + mangroves	7,828,315	741,185
Vulnerability + mangroves	5,290,370	1,559,876
**COMBINED CORAL REEF AND MANGROVE—highly vulnerable cells with at least 2500 ha of coral reefs and 1250 ha of mangroves**		
**Highly vulnerable cells ≤2 km from coast**	**Number of People**	**Ecosystem extent (hectares)**
Exposure + Sensitivity + mangroves + coral reefs	489,702	57,774
Vulnerability + mangroves + coral reefs	283,479	121,941

We calculated the number of people protected per hectare of either mangrove or coral reef in each country by dividing the number of people, as measured by the LECZ (S1 Text; S2 Fig), in the top 10th percentile of vulnerable cells in each country, by the number of hectares of each ecosystem extent per country.

**Fig 1:**
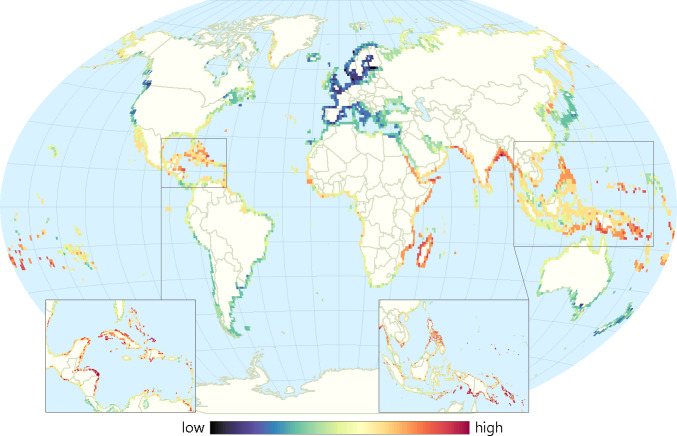
Vulnerability of coastal communities to sea-level rise and tropical storms. Insets highlight some of the most vulnerable coastal communities in the Caribbean and Southeast Asia.

Fig 1 depicts the top 10% of vulnerable cells globally and all figures are scaled up to 60-minute resolution (approximately 100km by 100km at the Equator), by taking the maximum vulnerability value, to best visualize broad trends, with the exception of insets, which show detail at the 10-minute analysis scale.

### Carbon co-benefits

We assessed the potential carbon storage co-benefit of mangrove ecosystems providing coastal protection services to vulnerable communities, by summing the estimated above and below-ground biomass and 1m of soil carbon as estimated by [60] that overlapped communities in the top 10^th^ percentile of vulnerability that contained at least 1250ha of mangroves (i.e. we calculated carbon in EbA hotspots, as defined above). We used the biomass estimate derived from Equation 5, which provided the best global estimates and report findings using their low and high belowground biomass estimate ranges [60].

### Ecosystem degradation/protection

As an estimate of the level of degradation to mangrove ecosystems globally, we used a 30 m resolution database of mangrove cover to estimate trends in mangrove area by country from 2000–2012 [61]. Similarly, to characterize potential degradation to coral reefs we used a 500 m resolution database of thermal and acidification potential to coral reefs in 2011 [51]. Though these two datasets report different measures–loss for mangroves and potential for thermal/acidification impacts for corals, we assume that both equate to potential degradation. This may result in an underestimate of mangrove degradation, as loss is only one possible type of degradation, and an underestimate of coral degradation, as thermal and acidification potential don’t include loss specifically. Because EbA hotspots and degradation data were at different resolutions, we collated degradation levels for any country that contained EbA hotspots in our analysis, as defined by either the top 10^th^ percentile countries as calculated by maximum or mean vulnerability in Table 3. Degradation levels were calculated at the entire country scale. To assess existing protection status of the relevant ecosystems, we overlaid the global extent of protected areas from the Protected Planet Database [62] with EbA hotspot cells to quantify the extent to which both coral reefs and mangroves in these areas are already protected.

**Table 3:** Countries with the highest vulnerability to sea-level rise and tropical storms (defined as top 10^th^ percentile mean and/or maximum vulnerability as calculated from this study), and their relative potential for coral reef acidification or thermal impacts, their percent mangrove change from 2000–2012, and their estimated carbon storage as calculated from other studies. Data for coral reefs are from [51]; data for mangrove change are from [61]; data for carbon storage are the conservative values from [60].

Country	% Coral Reefs at Risk	% Mangrove change 2000–2012	Mangrove C storage (Metric tons)
Cook Islands	11.75%	NA	NA
Turks and Caicos Islands	12.01%	0.00%	8,206
New Caledonia	13.55%	-0.60%	4,483,876
Eritrea	13.63%	-0.78%	82,520
Tonga	14.00%	NA	NA
Tuvalu	15.59%	NA	NA
Micronesia	16.80%	-0.43%	415,422
Somalia	25.32%	0.00%	623,448
Niue	27.01%	NA	NA
Solomon Islands	35.60%	-0.50%	20,434,559
Papua New Guinea	35.92%	-0.42%	213,771,039
Myanmar	39.15%	-8.42%	122,716,200
Fiji	40.56%	-0.19%	18,513,364
Indonesia	44.08%	-3.11%	1,222,287,244
Bermuda	53.99%	0.00%	NA
Mozambique	56.54%	-0.21%	55,298,827
Madagascar	60.38%	-0.27%	36,100,464
India	63.22%	-3.30%	9,458,516
Venezuela	68.73%	-0.50%	106,983,740
Honduras	76.42%	-2.00%	30,821,507
Dominican Republic	82.04%	-0.53%	5,176,471
Samoa	93.74%	NA	NA
Comoros	99.93%	0.00%	37,162
Saint Kitts and Nevis	100.00%	-20.00%	6,602
Pakistan	NA	-0.26%	514,233
Bangladesh	NA	-0.05%	71,924,116

The data we gathered to create the vulnerability index, calculate EbA hotspots, and determine carbon storage and ecosystem degradation come from different years, scales, and resolutions. Combining and downscaling them as we have done here may result in data loss and potential bias. For example, some adaptive capacity layers were at the country scale, despite there likely being differences within country. However, what we attempt to map are large-scale patterns of ecosystems that change rather slowly, reducing this possibility. Moreover, we weighed the importance of including as many influential factors as possible relating to coastal communities’ vulnerability as higher than matching perfectly temporal/spatial scales and resolutions. We emphasize, therefore, that our results are a coarse approximation at a global scale, and that finer-scale and on-the-groundwork is necessary to further investigate EbA potential.

## Results

### Vulnerability and EbA hotspots

**Fig 2:**
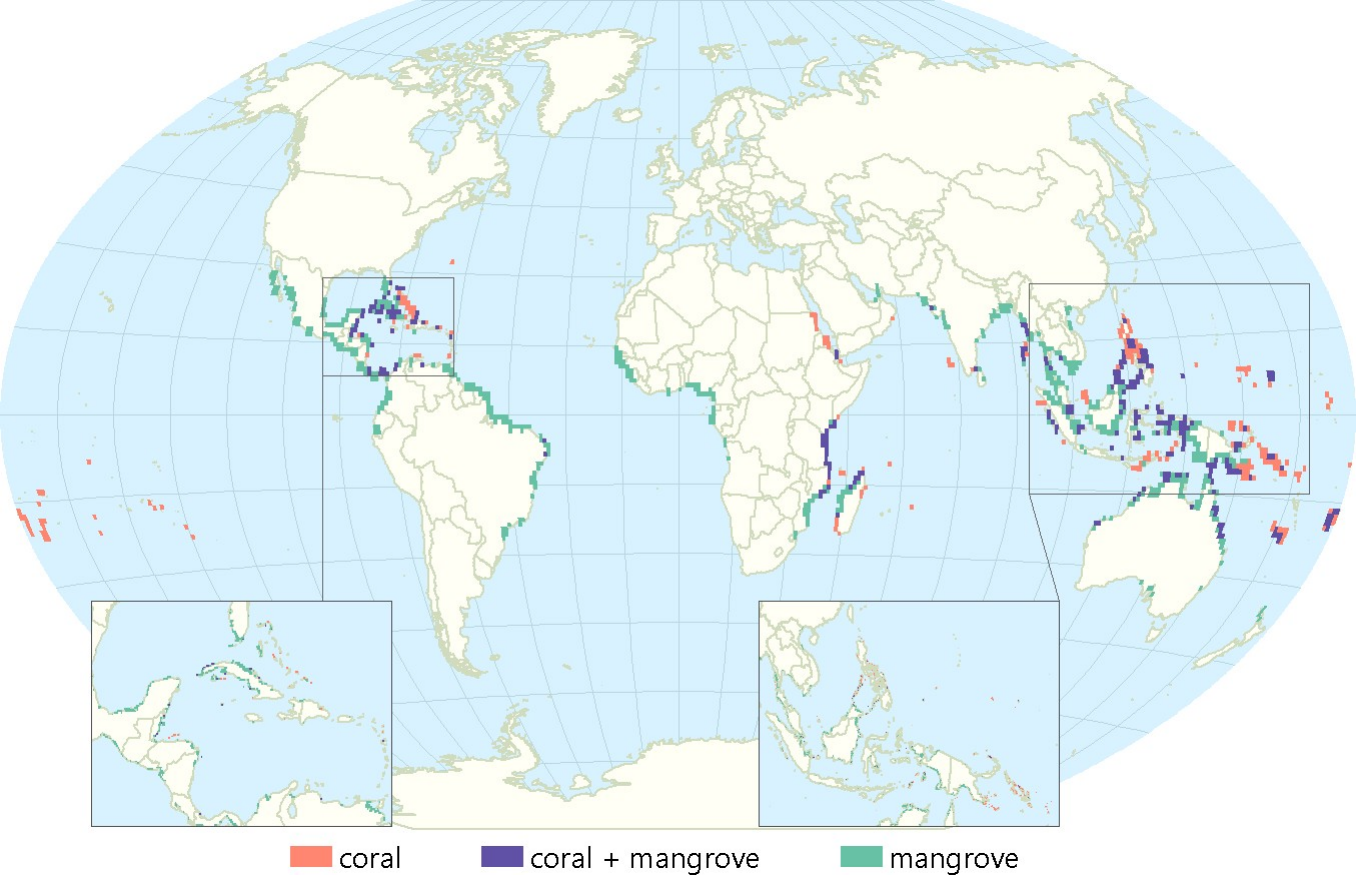
Global distribution of EbA hotspots categorized by ecoystem affording protection. Insets highlight some of the most vulnerable coastal communities in the Caribbean and Southeast Asia.

We find that at least 30.9 million people reside in areas highly vulnerable to SLR and storms (top 10% vulnerable cells within 2km of the coastline) (Fig 1; Table 2). Mangroves and coral reefs intersect with these vulnerable coastal populations across at least 1.6 million and 1.8 million hectares of the world’s coastline, respectively, potentially providing a coastal protection (adaptation) service to at least 5.3 million (mangroves) and 3.4 million (coral reefs) people (Fig 2). The densest populations receiving adaptation benefits (people protected per hectare) from mangroves are in India, the United States, and Ghana while the fewest people per unit area benefitting are found in Suriname, Nicaragua, and Gabon. The greatest amount of people protected per hectare of coral reefs are in South Africa, Singapore, China, and the United States while lower densities of people protected by coral reefs are found in Tokelau, the Marshall Islands, and Kiribati. This reflects the differences of population densities in these countries, rather than the importance of EbA for protecting those communities.

### Carbon co-benefits

Mangroves that intersect EbA hotspots store at least 896 million metric tons of carbon as aboveground and belowground biomass and soil carbon (Table 4). EbA hotspot countries that contain the highest carbon are Indonesia, Papua New Guinea, and Myanmar (Table 3). Of the EbA hotspot countries that have mangroves, St. Kitts and Nevis, Turks and Caicos, and Comoros store the least amount of carbon (Table 3). These rankings are driven by country size, with high carbon storage countries being some of the largest and low carbon storage countries being some of the smallest in area.

**Table 4:** Estimated aboveground, belowground, and 1m of soil carbon in EbA hotspots that contain mangroves. Low values are from [60]’s most conservative estimates and High values are from their most liberal estimates.

Estimated Carbon in Mangrove EbA Hotspots—highly vulnerable cells with at least 1250 ha of mangroves	
**Highly vulnerable cells ≤2 km from coast**	**Metric Tons**
Exposure + Sensitivity + mangroves (Low)	306,919,893
Vulnerability + mangroves (Low)	896,878,111
Exposure + Sensitivity + mangroves (High)	317,221,436
Vulnerability + mangroves (High)	935,372,222

### Ecosystem degradation/protection

**Fig 3:**
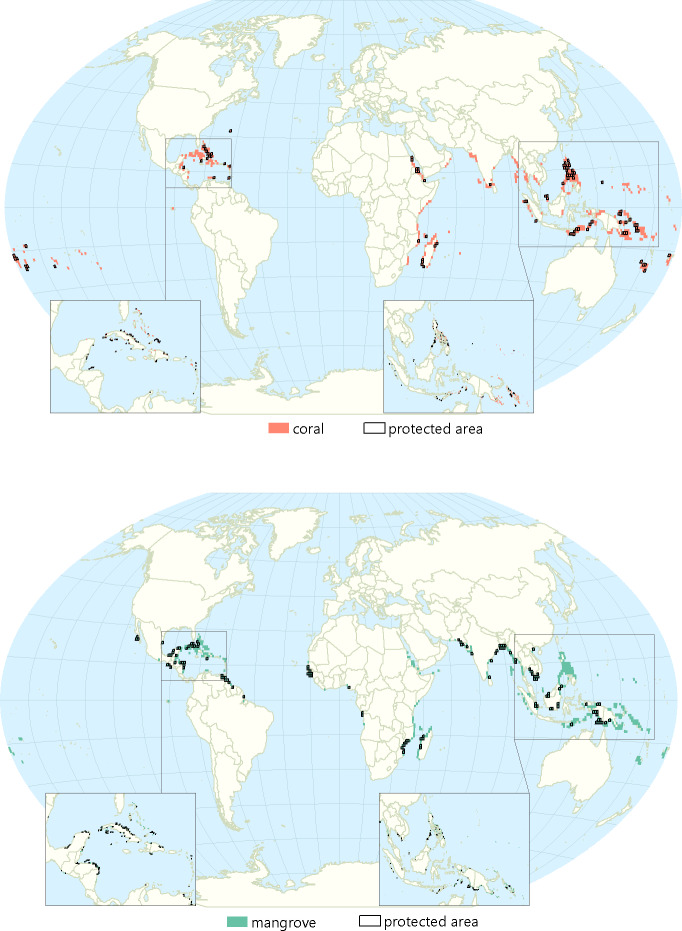
(Top). Mangroves likely conferring adaptation benefits to the most vulnerable coastal communities, and where they overlap with protected areas. (Bottom). Coral reefs likely conferring adaptation benefits to the most vulnerable coastal communities, and where they overlap with protected areas.

Thirty-eight percent of mangroves and 11% of coral reefs in EbA hotspots are protected (Fig 3). Of countries that contain EbA hotspots, Saint Kitts and Nevis, Myanmar, and India have experienced the biggest mangrove declines as a percent of their total mangrove area while Turks and Caicos, Somalia, Bermuda, and Comoros have not experienced mangrove declines (Table 3). Saint Kitts and Nevis, Comoros, Samoa, and the Dominican Republic are the countries that contain EbA hotspots with the highest proportion of their coral reefs at high risk while the Cook Islands, Turks and Caicos, and New Caledonia have the lowest proportion of their reefs at risk (Table 3).

## Discussion

Ecosystem-based adaptation (EbA) solutions provide promising potential for protecting people from the adverse effects of climate change across a range of adaptation contexts [63–65]. Despite mounting interest in EbA, implementation has mostly been in the form of individual site-specific projects, with little systematic assessment of its broader potential. Our analysis indicates that at minimum over 5 million people globally currently receive a potentially significant coastal protection service provided by mangroves and coral reefs, with the value of that service likely to grow under climate change driven increases in storm intensity and sea level rise. Though they provide this potentially crucial and widespread adaptation service, both mangroves and coral reefs face an array of threats that may degrade or destroy the service unless efforts are made to conserve these ecosystems.

Our findings expand on and comport with other studies that focused solely on exposure and sensitivity to calculate EbA potential [12,66]. For example, [66] used a sophisticated model to quantify where communities were receiving the largest benefits of flood avoidance from mangroves globally and found that Vietnam, India, Bangladesh, China, and the Philippines all receive the highest benefit. Similar to [66], [13] included GDP and did an economic analysis of potential monetary losses from coastal flooding. Both studies, therefore, highlighted countries such as China [58], the United States, the Netherlands and Japan [59], with high EbA potential that our study did not. This discrepancy is because our study highlighted those countries with the lowest adaptive capacity, while including GDP as a measure of potential benefit highlights those with high adaptive capacity. Our findings most closely mirror those of [12], because they focused solely on developing countries and our analysis, due to the inclusion of adaptive capacity, most frequently highlighted them as well. Findings from all of these studies underscore the enormous potential of EbA for helping to reduce both the economic and the human costs of coastal flooding, regardless of GDP.

### Potential for EbA to reduce risk to vulnerable people

The most vulnerable coastal communities are in developing countries, which are often characterized by high exposure, high sensitivity and low adaptive capacity. Low income communities with low governance, education, health, infrastructure, and access to markets (cooler colors in S3 Fig) stand to disproportionately benefit from adopting EbA solutions because such solutions may be cheaper to establish and maintain in many contexts [15] than built infrastructure. Given the economic and job insecurity in many of the most vulnerable communities, the potential for EbA to provide tourism, commercial/artisanal fishery, and other economic co-benefits could improve lives and provide livelihoods by providing for further economic and urban growth.

Our analysis of current ecosystems providing benefits to coastal populations assumes a simple spatial model of these benefits, without accounting for the fact that ecosystems provide relatively higher protection for those living nearest to coasts, and less protection for those further inland [35] and that ecosystem size, continuity, width and position on the landscape are all key factors in the magnitude of coastal protection ecosystems can provide [23]. Our goal was to provide a broad visualization for EbA potential rather than providing an in-depth analysis of the ways in which ecosystem protection varies with these important factors. Such analyses at smaller scales, such as [67] did for Brazilian cities will be important steps going forward to help regional and local adaptation managers optimize sites in which to pursue EbA.

### Policy recommendations

Our maps of coastal EbA hotspots, and their constituent maps of vulnerability and its components, provide multiple key insights to decisionmakers. Managers might look for areas with relatively low adaptive capacity and high exposure and sensitivity to floods and SLR, as depicted in dark red in Fig 1—these areas are clearly important places to maintain or enhance ecosystems. However, even in countries with relatively high adaptive capacity, using EbA may often be the most readily available and cost-effective option, and one which also benefits biodiversity and provides ecosystem services. Such areas of high adaptive capacity and high exposure and sensitivity may wish to employ EbA alongside sea walls, beach nourishing, levees, or other traditional hard engineering approaches to best prepare people for future storms and SLR. In contrast, areas highlighted in Fig 1 with the highest vulnerability and those in Fig 2, with ecosystems already present to help people adapt to climate change may have few options beyond EbA to help their coastal communities adapt to SLR and storms. Finally, in areas that have high sensitivity near mangroves and coral reefs, but which do not currently have high exposure, researchers and managers should project and track future exposure to coastal climate threats, and consider the maintenance of mangroves and coral reefs a hedge against uncertain future risks.

### Carbon storage co-benefits of using EbA

Ecosystems provide a whole host of services to people beyond adaptation. The additional benefits of protecting or restoring coastal ecosystems range from carbon storage and water filtration, to recreational opportunities and habitat for commercially important fish species. Our analysis suggests that mangroves in EbA hotspots are storing at least 896 million metric tons of carbon–equivalent to about 10% of global CO_2_ fossil emissions [68]. This means that investments in these systems for their adaptation benefits can also help mitigate the effects of climate change. Moreover, the mitigation values of these ecosystems [69] provide opportunities to leverage climate mitigation finance to make EbA investments more effective. This is especially important given that in the last few decades, an estimated one-third of mangroves, seagrass beds, and salt marshes have been lost to land-use change, invasive species, eutrophication, and development, among others [70].

### Ecosystem degradation/protection status

The fact that at least 38% of mangroves that intersect with EbA hotspots already have some level of protection is promising for the ability of people to benefit now and in the future from mangrove protection. However, greater levels of protection for mangroves, especially in countries with low adaptive capacity is a critical need for EbA solutions to reach their full potential. Mangrove protections are especially important in light of recent analyses, which show global yearly losses of mangroves of 0.16–0.39% [61]. Viable EbA solutions using mangroves will need to include protection of current mangrove cover, and could also incorporate mangrove restoration.

A little over 10% of coral reefs that coincide with EbA hotspots are protected, underscoring the need for more Marine Protected Areas (MPAs) to ensure protection from reefs in the future. It is promising that globally, protected areas are covering more of the planet [71], especially so for MPAs. Given recent research showing protected coral reefs are better able to withstand and recover more quickly from storms and bleaching [72], EbA solutions for communities living near coral reefs should consider reef protection.

### Future research needs

A key question for future research is the capacity of ecosystems to withstand climate change while still providing adaptation benefits to coastal communities. Some research suggests wetlands and mangroves may be able to migrate inward as seas rise, but only given relatively slow rates of rise and only if there are no infrastructural barriers to overcome [69]. Research clarifying this issue and adaptation plans that combine EbA with measures that may include assisting ecosystems to migrate inland as seas rise will both be integral as SLR progresses. Ocean acidification is also a major concern for coral reef ecosystems and underscores the need to combine adaptation strategies with aggressive mitigation to ensure ecosystems can continue to provide the adaptation and co-benefits they currently do. Another key area for future research is to quantify the amount of storm surge, wind, and sea level rise any given coastal ecosystem can withstand and still provide adaptation benefits to coastal communities. Further, studying how the surrounding landscape, ecosystem size, shape, and connectivity affect the ability of coastal ecosystems to provide adaptation services is equally crucial.

As resource use and human populations grow, ecosystem restoration is becoming one of the most important and widely used techniques to repair damage on afflicted ecosystems. Our findings provide added perspective to efforts like the Global Mangrove Alliance and its goal of restoration to increase mangrove extent 20% by 2030. Our analysis did not quantify the potential for restoring coastal ecosystems to help people adapt to climate change but rather focused on extant systems. Restoration may prove a crucial strategy for employing ecosystem-based adaptation approaches in the many areas where nature has been damaged. Although ecosystem recovery has been possible and timely in many cases [73], damaged ecosystems may not recover to their pre-disturbance functioning for a long time if ever [74,75]. Research on how long it takes for ecosystems to provide adaptation services after restoration is initiated, the level of protection restored ecosystems can provide relative to undisturbed ecosystems, and how resilient both undisturbed and restored ecosystems will be to continued anthropogenic or natural perturbations will help adaptation planners decide how to prioritize their efforts to maximize the protective benefits of both contemporary ecosystems and those of the future.

Although EbA is a relatively new concept, there has now been a variety of reviews highlighting other important needs to push EbA forward. A review of the EbA literature for urban ecosystems suggested more work is necessary to consider issues of equity and stakeholder participation for EbA and that research is often siloed, focusing on bio/geophysical aspects, rather than social and economic valuation [76]. Expanding on the idea of increasing the harmonization between disciplines, [77] urged a melding of the more commonly employed social-ecological systems EbA research with socio-technical systems EbA research. A consistently applied framework for addressing EbA options, that includes issues of equity, stakeholders, social, economic, bio/geophysical, and technical components would help push research forward.

## Conclusion

Our results provide broader context to complementary analyses [6,26] of vulnerability and EbA potential that were limited to specific regions and ecosystems. Our analysis should not be interpreted as suggesting that only areas with high vulnerability should consider implementing EbA approaches. Rather, the utility of identifying coastal vulnerability and areas where ecosystems can serve to help people adapt to climate change at the global scale lies in the ability to prioritize areas for further investigation and investment. Indeed, EbA are most often employed at local to regional scales and so local/regional factors (e.g., institutional capacity, tradeoffs between livelihoods and ecosystem conservation/restoration) that we were unable to include in our global analysis will play key roles in on-the-ground EbA deployment.

Arguments against the use of EbA sometimes invoke lost infrastructure development opportunities, especially in developing countries. However, urban coastal development and EbA need not be at odds. The majority of the world lives within 200km of the coasts, which means coastal development and urban planning are inevitable in these areas [10,11]. Including EbA in the list of options for these plans could help meet Sustainable Development Goals and adhere to the Sendai Framework for Disaster Risk Reduction, while also ensuring key ecosystem services like coastal protection are simultaneously provided to coastal communities. As initiatives like Wealth Accounting and the Valuation of Ecosystem Services (WAVES) push for economic accounting that includes the value of natural resources, valuing maintained or restored ecosystems should be considered as a way to manage human risk from natural disasters like hurricanes and could result in increased marginal benefits relative to other potential adaptation strategies. While investment in EbA has been growing, current investments are eclipsed by the untapped potential for more EbA solutions globally.

## Supporting information

S1 Table List of transformations used to improve normality of input variable distributions and adjustments for interpretation of high values. (DOCX)

S2 Table Numbers for less conservative estimates than reported in the main text Table 2. (DOCX)

S1 Fig Exposure to sea-level rise and tropical storms. Insets highlight some of the most vulnerable coastal communities in the Caribbean and Southeast Asia.
(DOCX)

S2 Fig Sensitivity index, also termed low-elevation coastal zone. Insets highlight some of the most vulnerable coastal communities in the Caribbean and Southeast Asia.
(DOCX)

S3 Fig Adaptive capacity. Insets highlight some of the most vulnerable coastal communities in the Caribbean and Southeast Asia.
(DOCX)

S1 Text [78–84]. (DOCX)
