# Supplementary material for: Global hotspots for coastal ecosystem-based adaptation
Source: PLoS One. 2020 May 29;15(5):e0233005. doi: 10.1371/journal.pone.0233005 (PMC7259744; doi:10.1371/journal.pone.0233005)
Supplement: S1 Table — (DOCX) [file pone.0233005.s001.docx]

S1 Table: List of transformations used to improve normality of input variable distributions and adjustments for interpretation of high values

| **Input variable** | **Transformation** | **Directional adjustment** |
| --- | --- | --- |
| Education | Johnson | Yes |
| Governance | Johnson | Yes |
| Income | Johnson | Yes |
| Travel | Johnson | No |
| Health | Logarithm | No |
| Impervious surfaces | Logarithm | Yes |
| Low elevation coastal zone | Power 1/5 | No |
| Sea-level rise | Logarithm | No |
| Storms | Logarithm | No |
