# Supplementary material for: Global hotspots for coastal ecosystem-based adaptation
Source: PLoS One. 2020 May 29;15(5):e0233005. doi: 10.1371/journal.pone.0233005 (PMC7259744; doi:10.1371/journal.pone.0233005)
Supplement: S2 Table — (DOCX) [file pone.0233005.s002.docx]

S2 Table. Numbers for less conservative estimates than reported in the main text Table 2.

| **VULNERABILITY** | ***Number of People*** | |  | |
| --- | --- | --- | --- | --- |
| **Number of people (<25 km from coast of top 10th percentile of vulnerability for 10 min cells)** | **20th percentile** | **50th percentile** |  |  |
| Exposure + Sensitivity | 772,981,747 | 1,139,691,110 |  |  |
| Vulnerability (Exposure + Sensitivity + Adaptive Capacity) | 308,842,672 | 700,236,952 |  |  |
| **Number of people (<2 km from coast of top 10th percentile of vulnerability for 10 min cells)** |  |  |  |  |
| Exposure + Sensitivity | 143,585,725 | 234,613,495 |  |  |
| Vulnerability - Exposure + Sensitivity + Adaptive Capacity | 59,373,434 | 143,544,359 |  |  |
| **CORAL REEF - highly vulnerable cells with or adjacent to at least 10 ha of coral reefs** | ***Number of People*** | | ***Ecosystem extent (hectares)*** | |
| **Highly vulnerable cells (<2km from coast)** | **20th percentile** | **50th percentile** | **20th percentile** | **50th percentile** |
| Impact + coral reefs | 35,279,312 | 58,772,877 | 3,037,722 | 6,525,558 |
| Vulnerability + coral reefs | 24,743,072 | 56,476,600 | 4,801,342 | 8,870,565 |
| **CORAL REEF - highly vulnerable cells with or adjacent to at least 2500 ha of coral reefs** | ***Number of People*** | | ***Ecosystem extent (hectares)*** | |
| **Highly vulnerable cells (<2 km from coast)** | **20th percentile** | **50th percentile** | **20th percentile** | **50th percentile** |
| Exposure + Sensitivity + coral reefs | 6,582,207 | 11,338,862 | 1,849,568 | 3,814,121 |
| Vulnerability + coral reefs | 5,807,879 | 10,773,505 | 3,039,402 | 5,135,814 |
| **MANGROVE - highly vulnerable cells with at least 5ha of mangroves** | ***Number of People*** | | ***Ecosystem extent (hectares)*** | |
| **Highly vulnerable cells (<2 km from coast)** | **20th percentile** | **50th percentile** | **20th percentile** | **50th percentile** |
| Exposure + Sensitivity + mangroves | 52,192,174 | 87,261,653 | 1,751,808 | 4,601,212 |
| Vulnerability + mangroves | 36,701,974 | 76,671,924 | 3,243,538 | 5,917,118 |
| **MANGROVE - highly vulnerable cells with at least 1250 ha of mangroves** | ***Number of People*** | | ***Ecosystem extent (hectares)*** | |
| **Highly vulnerable cells (<2 km from coast)** | **20th percentile** | **50th percentile** | **20th percentile** | **50th percentile** |
| Exposure + Sensitivity + mangroves | 9,367,385 | 16,880,900 | 1,325,321 | 3,633,661 |
| Vulnerability + mangroves | 7,830,630 | 15,672,821 | 2,631,854 | 4,687,364 |
| **COMBINED CORAL REEF AND MANGROVE - highly vulnerable cells with at least 10 ha of coral reefs and 5 ha of mangroves** | ***Number of People*** | | ***Ecosystem extent (hectares)*** | |
| **Highly vulnerable cells (<2 km from coast)** | **20th percentile** | **50th percentile** | **20th percentile** | **50th percentile** |
| Exposure + Sensitivity + mangroves + coral reefs | 23,666,716 | 38,275,827 | 587,723 | 1,229,099 |
| Vulnerability + mangroves + coral reefs | 16,709,069 | 38,918,985 | 950,005 | 1,537,201 |
| **COMBINED CORAL REEF AND MANGROVE - highly vulnerable cells with at least 2500 ha of coral reefs and 1250 ha of mangroves** | ***Number of People*** | | ***Ecosystem extent (hectares)*** | |
| **Highly vulnerable cells (<2 km from coast)** | **20th percentile** | **50th percentile** | **20th percentile** | **50th percentile** |
| Exposure + Sensitivity + mangroves + coral reefs | 688,848 | 1,168,919 | 111,638 | 222,881 |
| Vulnerability + mangroves + coral reefs | 594,361 | 1,255,656 | 193,756 | 302,943 |
