# Supplementary material for: Global hotspots for coastal ecosystem-based adaptation
Source: PLoS One. 2020 May 29;15(5):e0233005. doi: 10.1371/journal.pone.0233005 (PMC7259744; doi:10.1371/journal.pone.0233005)
Supplement: S1 Fig — Insets highlight some of the most vulnerable coastal communities in the Caribbean and Southeast Asia. (DOCX) [file pone.0233005.s003.docx]

| **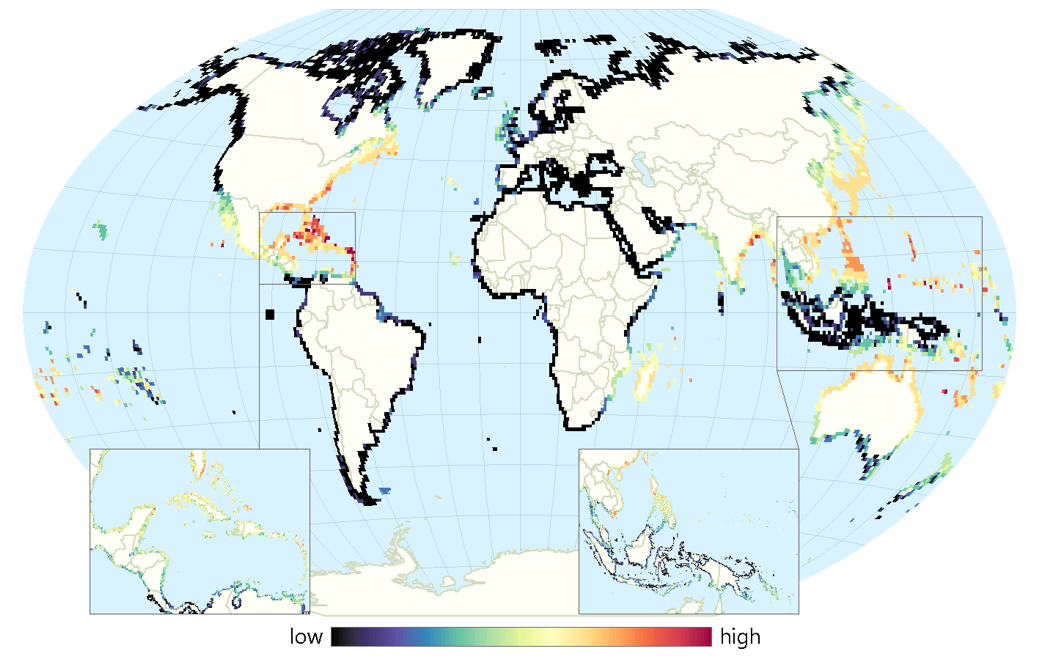** |
| --- |
| **S1 Figure.** Exposure to sea-level rise and tropical storms. Insets highlight some of the most vulnerable coastal communities in the Caribbean and Southeast Asia. |
