# Supplementary material for: Global hotspots for coastal ecosystem-based adaptation
Source: PLoS One. 2020 May 29;15(5):e0233005. doi: 10.1371/journal.pone.0233005 (PMC7259744; doi:10.1371/journal.pone.0233005)
Supplement: S3 Fig — Insets highlight some of the most vulnerable coastal communities in the Caribbean and Southeast Asia. (DOCX) [file pone.0233005.s005.docx]

| \| 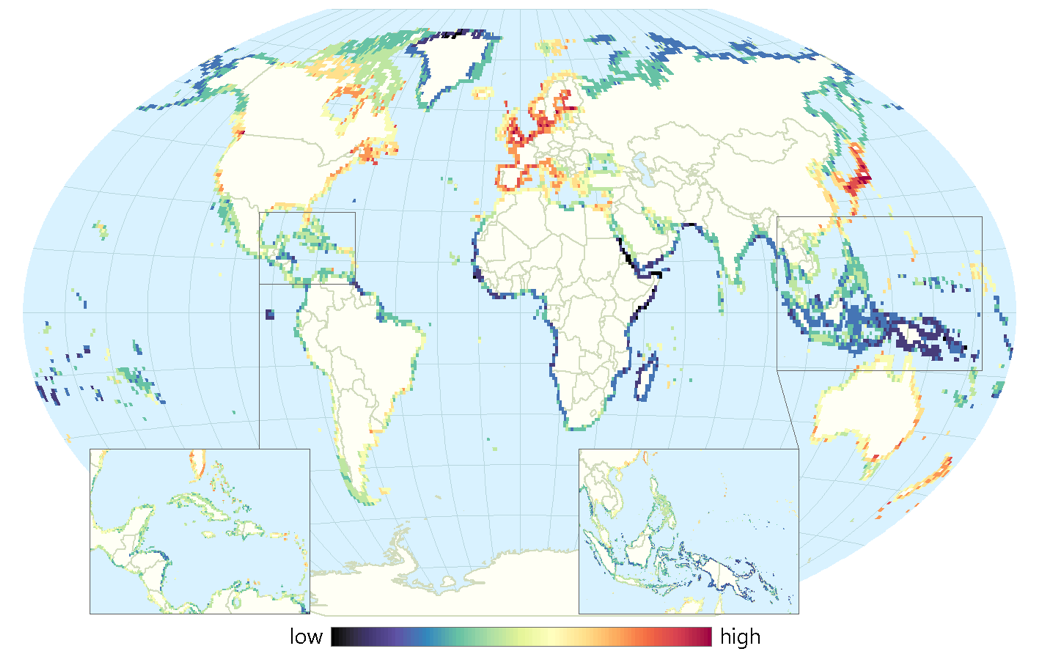 \| \| --- \| \| **S3 Figure.** Adaptive capacity. Insets highlight some of the most vulnerable coastal communities in the Caribbean and Southeast Asia. \| |
| --- | --- | --- |
