# Supplementary material for: Global hotspots for coastal ecosystem-based adaptation
Source: PLoS One. 2020 May 29;15(5):e0233005. doi: 10.1371/journal.pone.0233005 (PMC7259744; doi:10.1371/journal.pone.0233005)
Supplement: S1 Text — (DOCX) [file pone.0233005.s006.docx]

**Supporting information**

Prior to calculating indices for exposure, sensitivity, adaptive capacity and the full vulnerability index, we carried out two initial data manipulations. First, the distributions of all input variables were assessed and transformed to detect anomalies and improve normality. The latter ensured that the variables do not exert influence on the composite indices due to distributional features such as variance and skewness. Logarithmic, power and Johnson transformations (based on the Z family of distributions) were used to improve normality and harmonize variances, as appropriate for each index. Second, to obtain indices whose maxima indicate maximum vulnerability, any inputs whose interpretation was the reverse (impervious surfaces, income, education, and governance) were transformed by subtracting the data from their maximum value, respectively.

Index values were then calculated as follows: the transformed variables (S1 Table) were standardized on a scale from zero to one (together with the normality-improving transformations, this helps ensure that the variables’ contribution to the indices correlates with the formula used) and aggregated to the exposure, sensitivity and adaptive capacity indices. These ‘sub-indices’ were then standardized again and aggregated to the full vulnerability index.

*Exposure*

Coastal communities face a variety of climate-related threats including sea surface temperature increase, ocean acidification, and coastal flooding resulting from elevated runoff in coastal catchments upstream of coastal areas. Here, we focus on two key climate change-relevant exposures for which coastal ecosystems (specifically mangroves and coral reefs) are able to reduce risk, and where mapping at the global scale is feasible: tropical storms and sea-level rise (SLR).

*Storms -* Climate change-driven shifts in the frequency or intensity of tropical storms from the observational record remain uncertain [78]. While both theory and model projections suggests climate change will strengthen tropical storms [79], there are no global spatially explicit representations of potential future storm tracks or intensities available. We therefore used the National Oceanic and Atmospheric Administration's International Best Track Archive for Climate Stewardship (IBTrACS) [42] to map the frequency of historical and contemporary tropical storms. We used a filtered subset of the tracks including all records with wind speeds >34 knots (i.e. those above ‘tropical depression’ intensity), over the period 1900­–2013, and buffered by 50 km, providing a conservative estimate approximating the mean radius of maximum wind based on IBTrACS data. We then calculated frequency of storm events within every coastal grid cell. As our indicator of future climate change-driven exposure to tropical storms, we assume that future storm frequency and intensity will correlate with historical frequency and intensity. This is a reasonable assumption, since our exposure index makes no attempt to quantify actual frequency or intensity; it simply represents an index of relative risk among coastal grid cells and regions globally.

*Sea-level rise -* In order to characterize future SLR, we used a global dataset that maps an anticipated 1 m rise in sea level on a global grid of 1 km resolution [41]. As with our storm indicator, our intent is to illustrate relative, not absolute, risk between regions; hence, the precise magnitude of SLR and time period over which it occurs is not critical to our analysis (with both being highly uncertain). We chose 1 m since research suggests that sea levels will increase anywhere from 0.5 to 2m above present levels by 2100 [80]. In order to convert the binary SLR dataset, which defines whether a 1 km cell is inundated or not to a relative indicator of SLR risk on a continuous scale, we calculated the percentage of 1 km cells at risk of inundation within each 10-minute coastal grid cell.

*Integrating exposure layers*

To produce a single spatially explicit global map of exposure, we normalized both our SLR and tropical storm indicators from 0 to 1, calculated a simple weighted average of (SLR*0.33) and (storms*0.67) for each coastal grid cell, then re-normalized to generate a composite index of relative exposure [81,82].

*Sensitivity*

We characterized human populations as being sensitive to climate change exposure(s) according to their density, proximity to the coast, and elevation [83]. We used simple thresholds for proximity to the coast (2 km or less) and elevation (15m or less) to define the ‘low elevation coastal zone’ (LECZ). Within this zone, sensitivity was defined by the LandScan [43] population density dataset, using raw population density normalized between 0 and 1 based on the highest density value within the global LECZ.

*Adaptive Capacity*

Adaptive capacity represents "the capabilities, resources and institutions of a country or region to implement effective adaptation measures" [40]. It is typically measured with a variety of socioeconomic metrics meant to serve as indicators of the resources available to a country or region to adapt to climate change. For example, [84] define adaptive capacity as a function of eight indicators including availability of technology, resource availability and distribution, institutional structure, human and social capital (including education and personal security), risk-spreading mechanisms, decision-maker ability to manage risk/information, and public perception of the source of stress and the significance of exposure. Here, we used six indicators representing key dimensions that have been identified playing major roles in adaptive capacity [40] and are available at the global scale – i) governance; ii) income; iii) education, iv) health, v) access to markets and vi) infrastructure (Table 1). We normalized each indicator and calculated adaptive capacity as the mean across the six indicators for each pixel (S3 Figure). We applied no weighting factors since at global scale we could find no simple, consistent, defensible justification for placing more importance on any single dimension of adaptive capacity.

*Sensitivity analyses*

We tested several variations of the above approach to assess the validity and robustness of the indices. We tested weighting storms and sea-level rise equally but found the 67:33 split better suited to capture the acute impacts of destructive storms on coastal regions. While further modifications can be made and tested, mapping and expert assessment of the different sensitivity scenarios confirmed the choice of index calculation method presented in the paper.
